# Fast volumetric registration method for tumor follow‐up in pulmonary CT exams

**DOI:** 10.1120/jacmp.v12i2.3450

**Published:** 2011-02-02

**Authors:** José Silvestre Silva, João Cancela, Luísa Teixeira

**Affiliations:** ^1^ Department of Physics Faculty of Sciences and Technology University of Coimbra Portugal; ^2^ Instrumentation Center Faculty of Sciences and Technology University of Coimbra Portugal; ^3^ Department of Radiology University Hospital of Coimbra Portugal

**Keywords:** 3D image registration, similarity metrics, lung tumors, computed tomography

## Abstract

An oncological patient may go through several tomographic acquisitions during a period of time, needing an appropriate registration. We propose an automatic volumetric intrapatient registration method for tumor follow‐up in pulmonary CT exams. The performance of our method is evaluated and compared with other registration methods based on optimization techniques. We also compared the metrics behavior to inspect which metric is more sensitive to changes due to the presence of lung tumors.

PACS numbers: 87.57.nj; 87.57.Q‐; 87.57.N‐

## I. INTRODUCTION

Computed tomography (CT) is a medical imaging tool that allows for volumetric data acquisition. With high‐resolution computed tomography and, more recently, with multirow spiral CT, it is possible to obtain very thin slices of the thoracic region, having a high resolution and contrast between lungs and nearby structures, offering more diagnostic options and a better diagnostic quality. As a result, the large number of slices will also increase the time needed for data reading by the radiologist. Therefore, computer aid is necessary in order to increase the level of efficiency and quality in the diagnostic workflow.^(1)^

Image registration is a method for matching two or more images of the same object taken at different times, and/or by different modalities. It geometrically aligns two images: the reference and sensed images. To register two images, it is necessary to find a transformation so that each pixel in a single image can be mapped to a pixel in the second.^(^ ^2^ ^–^ ^4^ ^)^ The image registration is used in several clinical scenarios. For instance, consider two images taken of a patient using different medical modalities or comparing two CT exams from a patient (one taken some time ago and the other taken today), to identify the differences between the two images in a follow‐up study of an oncological patient. Despite the fact that this identification can be done interactively by the radiologist, there is always the possibility that some features could be missed in a follow‐up of oncological patients.^(^ ^5^ ^–^ ^9^ ^)^

In the literature we have found some work in this area. Betke et al.^(10)^ developed an automated registration method for the chest CT images based on anatomical landmarks: the trachea, carina, vertebrae, sternum and spine. They used an iterative lung surface registration method based on minimizing Euclidean distances. The method automatically detects and compares the lung nodules obtained at different times. The registration is performed point‐to‐point together with affine transformations (scale, shear, rotation and translation), followed by an iterative process of alignment. The selected similarity metric was the correlation and the sum of the squares of the differences between the two CT volumes. They used 10 exams from two patients and concluded that the metric used shows a proper alignment of the nodule, that the best metric is the correlation coefficient, and that the best alignment of lung surface can be obtained performing another registration, using the search of the nearest point.

Blaffer et al.^(11)^ studied the precision and computation time of a rigid body using an affine and a spline‐based elastic registration approach on the full data volume. They compared the results to an affine registration that was preceded by a segmentation of the lung, using a downsized CT exam. One of the methods compared was based on B‐spline using six control points in each direction. The reducing of resolution was done from 512×512×250 to 32×32×32 voxels, followed by the determination of the matrices of rigid or affine transformations. The metric used was the sum of the squared differences in grey levels. This work involves the observation of patients with lung nodules, their detection and quantification. They concluded that the registration with affine transformations is slightly better than the registration with rigid transformations, and both are much quicker than the elastic registration.

Chen et al.^(12)^ presented a 2D‐3D image registration method for automatic pretreatment validation in radiotherapy. They implemented a hybrid cost function and a quick search method. Their work allowed for a better usage of radiation, decreasing the radiation doses in healthy tissues. Christensen et al.^(13)^ described a relationship between tracking lung motion using spirometry data and image registration of consecutive CT image volumes over multiple breathing periods. They modeled the motion of the lung using a small deformation linear elastic model, and demonstrated that the lung does not expand uniformly during the breathing period, but rather expands and contracts locally at different rates during inhalation and exhalation.

Dougherty et al.^(14)^ proposed a registration method for CT exams. Initially the translation is estimated, then the rotation and finally the values of these two transformations are used to start a transformation local vector for each lung, between the reference exam and the exam under study, to process the subregions of the image. They computed the sum of square of the differences to estimate the evolution of the registration method. Ten exams are used, and for two exams the final value of the correlation was 95%. Due to insufficient memory, the entire volume of the lung was not registered but only small regions of interest. El‐Baz et al.^(15)^ proposed a new methodology for 3DCT data registration which is nonrigid and involves two steps: a global target‐to‐prototype alignment of one scan to another using the learned prior appearance model, and a local alignment in order to correct for complex deformations. An affine transformation that globally registers a target to a prototype was estimated by the gradient ascent‐based maximization of a special Gibbs energy function.

Fung et al.^(16)^ compared two image fusion techniques during radiation release for cancer patients. The occurrence of unexpected translations and rotations led to the conclusion that the fusion through the visual alignment is more robust in some cases than the registration with control points; therefore, this was the elected method to study the patient position. Hong et al.^(17)^ presented an automated segmentation and registration method to detect and compare nodules in sequential chest CT scans. The registration is performed in four steps: pulmonary segmentation; the generation of a 3D distance map to evaluate the hierarchical surface registration that is used to improve the initial alignment; the manual detection of nodules; followed by matching with the smallest Euclidean distances. This method demonstrates a good accuracy in the extraction of the lungs.

Matsopoulos et al.^(18)^ proposed an automatic elastic registration method applied on thoracic CT exams of patients diagnosed with non‐small‐cell lung cancer. In a previous study, they didn't use nonrigid transformations to correct the lung movements; however, in this study, a logarithmic function was used to perform the elastic registration, showing better results for deformations due to breathing. Their methodology was compared with a method based on surface registration using rigid transformation and the authors concluded that their method produces better results.

Pluim et al.^(19)^ conducted a review of medical images registration with special attention to mutual information similarity metric. They concluded that mutual information is a metric frequently applied in registration methods, but other metrics produced better results, especially for registration methods using images from MRI of the brain. The authors also concluded that the mutual information is not the most suitable for fine structures (example: images of retina) or for combining MR with ultrasound. Rodeski et al.^(20)^ proposed a method for 3D registration of lung surfaces to determine the scaling parameter, using two different approaches: the first method uses lung surfaces and the second uses lung volumes. The method is performed twice to take into account the variations in volume due to breathing. They used affine transformations and for the similarity metric, they selected the sum of the square of the differences. The scale parameter is determined using the center of mass from the lungs. The final results were good, but a local registration is needed in order to account for the expansion of the lungs due to respiration.

Stewart et al.^(21)^ presented a hybrid registration method applied to the registration of images from the retina and also to the 4D registration of images from the lungs. They used a variant of the registration method (with nearest point search) for images of the retina and another method for the lungs. The control points from the retina were automatically defined as the center of vessels along the retina while, in the lungs, the division of the images was performed in small subregions and by analyzing the gradient of each subregion in order to find out the ones that have the highest gradient, according to the predefined information. These studies do not show any quantitative results and the experiences were performed in a small number of CT exams.

West et al.^(22)^ examined the problem of deformable registration of the abdomen. They created a model for the respiratory motion of abdominal organs because the deformation of the lungs during the respiratory cycle can lead to the movement of other organs (liver, kidney, etc.). They used twenty‐one control points to align the images and the transformations were based on B‐splines. Initially the axial resolution of the images was reduced to 64 pixels and the first registration was performed. The process was repeated for an axial resolution of 128 pixels. A new metric that depends on the position of control points and its intensity was introduced. This metric consists of the addition of normalized sum of the square of the intensity difference with the sum of the squared position differences between control points. They concluded that the traditional method based on intensity is insufficient and that their method is not sufficient for all cases.

Zheng et al.^(23)^ proposed a new method that simultaneously segments and performs the registration of the lungs and tumors in various CT exams. This method uses B‐splines to model the deformation of the lung, while a rigid transformation is introduced in the tumor to preserve its volume and shape. They used the sum of the squared differences as the similarity metric. Several control points are set within the tumor to form the control mesh and, therefore, it is assumed that the tumor follows the same rigid transformation as the control mesh.

In the following sections, we describe our methodology for the intrapatient registration of thoracic CT exams with the objective of pulmonary tumor discrimination in follow‐up studies of oncological patients.

## II. MATERIALS AND METHODS

The proposed method for the volumetric pulmonary registration (TDM) between two CT exams consists of three steps: starts with the noise reduction and segmentation of the lungs, followed by a preliminary alignment of the CT exams based on center of mass of the pulmonary region in each exam. Then, using a downsized version of the original image, the registration is performed using each transformation separately. Also in this work, we searched for the best metric, sensitive to changes in CT exams due to the presence of lung tumors. For comparison purposes, a traditional non optimized registration method ™ and also other methods based on the Nelder‐Mead simplex (SM) and the pattern search (PSM) optimization techniques were implemented.^(^ ^24^ ^–^ ^26^ ^)^

### A.1 Preprocessing

The images from high‐resolution multirow spiral CT are sensitive to noise; it can contribute negatively to the lung's segmentation. After comparing several denoising filters, we selected the geometric mean filter. In this filter, each pixel is the result of the product of all gray levels in a m×n neighborhood raised to the power of 1/(m×n). This filter tends to maintain more detail than the arithmetic mean filter.^(27)^

The pulmonary regions are identified using a previously developed^(^ ^28^ ^,^ ^29^ ^)^ and validated algorithm.^(^ ^9^ ^,^ ^30^ ^)^ Finally, in this pre‐alignment step, a translation is performed based on the differences between the center‐of‐mass coordinates of the 3D pulmonary region from each CT exam, in order to align both mass centers, allowing for a fast registration of pulmonary CT exams.

### A.2 Main registration

**Figure 1:**
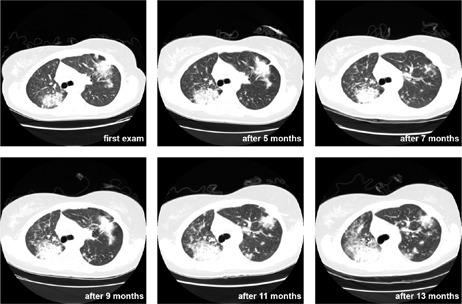
Images near carina level from CT exams of patient I, acquired in six different moments: at first exam (when the tumor was found) and other five exams acquired at 5, 7, 9, 11 and 13 months after the first exam.

The registration of two images can be understood as the determination of spatial alignment between images. Its applications includes: combining images of the same subject from different modalities, aligning temporal sequences of images to compensate for the subject's movement between scans, and image guidance during interventions.^(6)^ The proposed method performs the registration between 3D images taken from the same patient at different moments (see Fig. 1).

We consider affine transformations as the only relevant transformation for the registration of pulmonary CT exams and tumor follow‐up.^(11)^ Why affine transformation? The aim of our method is to compare two CT exams acquired on different days from the same patient with a lung tumor. The first logical choice would be deformable registration as B‐spline registration, optical flow registration, thin‐plates deformation, Cauchy‐Navier splines transformation, among others.^(5)^ As the deformable transformation may strongly change in shape, the object under study may be totally deformed to be coincident with the reference object, having lost the local differences between them. If a deformable transformation was used in the registration of CT exams showing a lung tumor from a patient who is under treatment, the differences in shape and volume of the tumor, when the two CT exams are aligned, might be almost null due to the deformable transformation used. Our main objective of the transformation is to globally align the two pulmonary exams, but this alignment should not modify significantly the shape and volume of the tumor. For this reason the right choice is not a deformable transformation, but the affine transformation.

In traditional registration methods ™, all translations are iteratively searched, and for each translation all rotations are searched, and so on; processing a 3D image becomes a very tedious process. In order to overcome this limitation, we propose a registration method that performs each transformation individually.

The first step is the search for the best translation; then, holding this best value, it searches for the best rotation over x‐axis. Holding these two best values, it searches for the best rotation over y‐axis, and so on – obtaining the best values for each transformation in a much faster approach than in traditional 3D registration methods. In the second step, it uses the best values found in the previous step as the starting point, and then repeats the same procedure described in the first step, searching for new best values. It searches for a new best value changing only translation; holding this new best value, it searches for the new best value for rotation, and so on, achieving a final best value for each transformation.

Defining boundaries for the transformations are very important in reducing processing time. We start by computing the width of the CT exam and use 1/8 of the width as the step for searching the best translation, over the x‐, y‐ and z‐axes in a range from −¼ of the exam width, to +¼ of the exam width. After searching along these axes, the best value is found comparing all values computed for the similarity metric. The best translation is identified and using this value as the starting point, a new search is performed in a range from −1/8 of the exam width to +1/8 of the exam width centered at the starting point, using a step of 1/16 of the exam width, which is half of the previous used step. This procedure is repeated until the step reaches the value lower than one voxel. Similar procedures are used for rotation in a range between −15° and +15° with a step of 7.5°, scale using a range of 1±0.14 with a step of 0.07, and for shear in a range of ± 0.14 with a step of 0.07. With this procedure, the best values are located in a faster way than the traditional registration methods.

### A.3 Normalized similarity metrics

Several normalized figures of merit, also known as normalized similarity metrics, were used to quantify the differences between the original image and the image under analysis.

As most figures of metric^(^ ^6^ ^,^ ^31^ ^–^ ^33^ ^)^ are not normalized and to compare the results among them, we performed their normalization, redefining the metric expressions so that they produce values between 0 (for two different images) and 1 (for two coincident images). We used the following metrics: the normalized sum of square of the differences (nSSD), the normalized sum of the absolute differences (nSAD), the correlation (R), the normalized disparity (nD), normalized joint entropy (nH), and the normalized mutual information (nMI).

(1) $$nSSD(A,B)=1-\frac{1}{N_{1}}\sum_{i}^{X}\sum_{j}^{Y}\sum_{k}^{Z}{\left[A(i,j,k)-B(i-m,j-n,k-o)\right]}^{2}$$

(2) $$nSAD(A,B)=1-\frac{1}{N_{2}}\sum_{i}^{X}\sum_{j}^{Y}\sum_{k}^{Z}\left|A(i,j,k)-B(i-m,j-n,k-o)\right|$$

(3) $$R=\frac{\sum_{i}^{X}\sum_{j}^{Y}\sum_{k}^{Z}A(i,j,k)\times B(i-m,j-n,k-o)}{\sqrt{\sum_{i}^{X}\sum_{j}^{Y}\sum_{k}^{Z}A{\left(i,j,k\right)}^{2}\times \sum_{i}^{X}\sum_{j}^{Y}\sum_{k}^{Z}B{\left(i-m,j-n,k-o\right)}^{2}}}$$

(4) $$nD(A,B)=1-\frac{1}{N_{3}}\sum_{i}^{X}\sum_{j}^{Y}\sum_{k}^{Z}A_{l}(i,j,k)⊕B_{l}(i-m,j-n,k-o)$$

(5) $$nH(A,B)=N_{4}+\sum_{a\in A}\sum_{b\in B}\rho_{AB}(a,b)\log_{2}[\rho_{AB}(a,b)]\;\;\;\;\;\;\;with\;\;\;\;\;\rho_{AB}=\frac{h_{AB}}{N}$$

(6) $$\begin{array}{l} nMI(A,B)=N_{5}+\frac{2MI(A,B)}{H(A)+H(B)}\;\;\;\;\;\;with\;\;\;\;\;H(A)=-\sum_{a\in A}\rho_{A}(a)\log_{2}\left[\rho_{A}(a)\right]\\ \;and\;\;\;\;\;MI(A,B)=\sum_{a\in A}\sum_{b\in B}\rho_{AB}(a,b)\log_{2}\frac{\rho_{AB}(a,b)}{\rho_{A}(a)\rho_{B}(b)} \end{array}$$

where $N_{1}=X\times Y\times Z\times I_{\text{max}}^{2};N_{2}=X\times Y\times Z\times I_{\text{max}};N_{3}=X\times Y\times Z;N_{4}=175/100;N_{5}=2;$ A and B are two images; i, j and k are the coordinates on the image; m, n and o are the displacement values to the reference image; $\rho_{\text{AB}}(a,b)$ is the joint intensity probability computed from joint histogram $h_{\text{AB}}(a,b)$ of the images A and B; $\rho_{A}(a),\rho_{B}(b)$ are the intensity probabilities computed from the images histograms of images A and B, respectively, $I_{\text{max}}$ is the maximum intensity of the images as $I_{\text{max}}=\text{max}(A,B);A_{l}$ and $B_{l}$ are binary images computed as $A_{l}=A>(I_{\text{max}}/2)$ and $B_{l}=B>(I_{\text{max}}/2);$ the operation ⊕ is the XOR operation, X and Y are the image size along the two axial axes and Z is the image size along the longitudinal axis.

**Figure 2:**
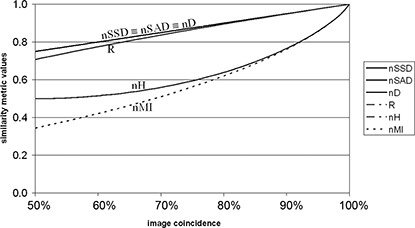
Behavior of normalized figures of merit, for two identical images that were misaligned. In xx axis, the pixel percentage in the coincident region of both images.

For nH and nMI it is assumed that more than 50% of the CT exams are in common region. This is true in most cases as the pulmonary region is in the central region of CT exam, so more that 50% of the voxels that belong to the pulmonary region in the exam from patient A are also in the pulmonary region of the CT exam from patient B. For successful application of previous normalized metrics, the images to be registered should be normalized, with grey levels between 0 and 1. (Fig. 2)

With the purpose of comparing the normalized figures of merit, a preview of their behavior is represented in Fig. 2, and will be discussed in next section.

### A.4 Other optimized registration methods implemented

The performance of the proposed method (TDM) was compared with two optimized methods that were also implemented, using the Nelder‐Mead simplex optimization technique and the pattern search optimization technique.^(^ ^24^ ^–^ ^26^ ^)^ These two methods are abbreviated as SM and PSM, respectively.

The SM optimization technique^(24)^ is a direct search method for multidimensional unconstrained minimization. Without any derivative information, a scalar‐valued nonlinear function of n real variables using only function values is minimized. This technique preserves at each stage a nondegenerate simplex, a volume different from zero in n dimensions which is the convex surface of n+1 vertices. It starts with a simplex, specified by its n+1 vertices and the related function values for each iteration. At least one test point is calculated, as well as the function values.

The PSM optimization technique^(^ ^25^ ^,^ ^26^ ^)^ is also a direct search technique and uses the function from a prearranged pattern of points fixed around the current best point, using shifts that guarantee determined minimal conditions in order to ensure the strong performance of the method. This process is repeated with the pattern centered on the new best point whenever certain minimal conditions are ensured. The reduction of the size of the pattern occurs and the function is sampled once again.

## III. RESULTS & DISCUSSION

We begin this section by describing the dataset and the working conditions. Then we present the results from the comparison of our method (TDM) with three other registration methods: the traditional method ™ and two other methods (SM and PSD) based on the simplex and the pattern search optimization techniques. The results include the correlation values, processing time and number of iterations executed, for each method.

To further study the registration method behavior, using CT exams with lung tumors and in some CT exams that did not have tumors, we created an artificial tumor.

All these results allow us not only to choose the best registration method but also to explore the behavior of normalized similarity metrics when used in registration of pulmonary CT exams to follow‐up lung tumors.

The results were computed on a desktop computer Intel Core I7 Quad 950 (Intel Corporation, Santa Clara, CA) using Windows 7 with MATLAB 2009a (The MathWorks, Natick, MA). During the developing stage, the methods were implemented using parallel processing (parallel for loops) on multiple cores, to optimize the performance of the tasks sent to the four cores of the I7‐950 processor.

### A.1 Dataset

Our dataset has a total of 96 CT exams from 20 patients (most of them have lung cancer and are undergoing therapy), with axial size of 512×512 pixels and most of the exams are volumetric CT exams (slices are adjacent to its neighboring slices). All CT exams were acquired at total lung capacity (maximum inspiration). Detail information, including voxel dimensions, average number of slices and relative acquisition date, are included^(^ ^3^ ^,^ ^4^ ^,^ ^34^ ^)^ in Table 1.

**Table 1:** Detail information of pulmonary CT exams under analysis.

			*Voxel Dimensions (mm^3^)*				
*Patient*	*Scanner*	*Average Number of Slices*	*X*	*Y*	*Z*	*First CT Acquisition*	*Additional CT Acquisitions*
**A**	(a)	86	1.36	1.36	5	$1^{\text{st}}$ exam	after 1, 3, 4 and 6 months
**B**	(a)	87	1.28	1.28	5	$1^{\text{st}}$ exam	after 2 and 3 months
**C**	(a)	90	1.28	1.28	5	$1^{\text{st}}$ exam	after 2 and 3 months
**D**	(a)	81	1.30	1.30	5	$1^{\text{st}}$ exam	after 2, 3, 4, 5 and 6 months
**E**	(a)	93	1.28	1.28	5	$1^{\text{st}}$ exam	after 2 months
**F**	(a)	86	1.28	1.28	5	$1^{\text{st}}$ exam	after 2 months
**G**	(a)	111	1.28	1.28	5	$1^{\text{st}}$ exam	after 2 and 4 months
**H**	(a)	90	1.38	1.38	5	$1^{\text{st}}$ exam	after 1 and 5 months
**I**	(b)	65	0.64	0.64	5	$1^{\text{st}}$ exam	after 5, 7, 9, 11 and 13 months
**J**	(b)	70	0.76	0.76	5	$1^{\text{st}}$ exam	after 2, 3, 5 and 6 months
**K**	(b)	55	0.75	0.75	5	or 7.5	$1^{\text{st}}$ exam after 2, 3, 5, 7, 8,10, 12, 14, 16, 18, 20 and 22 months
**L**	(b)	81	0.76	0.76	5	or 7.5	$1^{\text{st}}$ exam after 1, 3, 6, 9, 12, 13, 14, 16, 18, 19, 23, 25 and 27 months
**M**	(b)	57	0.75	0.75	5	or 7.5	$1^{\text{st}}$ exam after 3, 7, 11, 12, 13, 14, 16 and 18 months
**N**	(b)	64	0.65	0.65	5	or 7.5	$1^{\text{st}}$ exam after 1, 3 and 5 months
**O**	(b)	81	0.66	0.66	5	or 7.0	$1^{\text{st}}$ exam after 6, 13, 17, 20 and 27 months
**P**	(c)	72	0.70	0.70	7	$1^{\text{st}}$ exam	after 6, 10 and 16 months
**Q**	(c)	124	1.42	1.42	2.5	$1^{\text{st}}$ exam	after 7 months
**R**	(c)	119	1.33	1.33	2.5	$1^{\text{st}}$ exam	after 3 months
**S**	(c)	66	1.35	1.35	1.25	$1^{\text{st}}$ exam	after 7 months
**T**	(c)	123	1.54	1.54	2.5	$1^{\text{st}}$ exam	after 6 months

(a) Toshiba Asteion; (b) GE Light Speed 16; exams downloaded from National Cancer Imaging Archive (www.nci.nih.gov); (c) GE Light Speed VCT.

**Figure 3:**
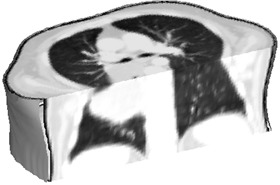
3D image of thoracic region from CT exam of patient B, near carina level, where white mass corresponds to pulmonary tumor.

To reduce the processing time, for each exam and using projection of maximum intensity, we located the smallest rectangular cuboid (parallelepiped, where all angles are right angles) that contains the lungs, identified the pulmonary region and the volume of the exam is reduced, discarding voxels outside the rectangular cuboid. Also, to decrease computational time, each slice was downsized from 512×512 to 256×256 or 128×128. The longitudinal dimension of CT exam is kept constant. After this procedure, a new 3D binary volume corresponding to the pulmonary region is built (see Fig. 3)

The 3D images produced by our method, after processing a CT exam, show a clear view of the lung tumor, as represented in Fig. 3.

The registration method, proposed in this work, was applied with success, in intrapatient registration of thoracic CT exams, tested in exams of 20 patients with oncologic diseases. In Fig. 1, we show the output of our method, in a 2D view. These images are from a follow‐up study of an oncologic patient and were acquired through a period of 13 months. Our method correctly aligns the 3D exam, allowing the radiologist to observe in 3D or 2D images the evolution of a small white mass in the lung.

During the development stage, we used two exams from one patient and another two exams from a second patient (these patients are not included in the Table 1). An expert radiologist supervised the output of the TDM, giving feedback to the developers, to achieve an optimal registration not only to obtain the best value for the similarity metric, but also to ensure that the registration is correct in a visual inspection of the pulmonary CT exams.

### A.2 Comparison of registration methods

In this section, we compare the results of four 3D pulmonary registration methods, performing the intrapatient registration of two exams. The results from exams downsized to 256×256×n and downsized to 128×128×n (where n is the original number of sections) were not so different; therefore, for the remaining comparisons, we used exams downsized to 128×128×n.

**Figure 4:**
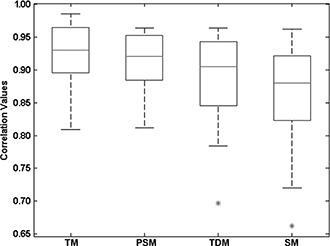
Box‐plot graphic of correlation values computed using four registration methods in exams from 20 patients. The star (*) indicates the outliers.

**Figure 5:**
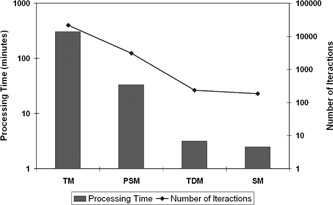
Average processing time and number of iterations computed using four registration methods in exams from 20 patients.

Using the exams shown in Table 1, we performed the registration of each patient's first exam with their second exam, for each patient, in a total of 20 patients. The registration methods used were TM, PSM, TDM and SM, implemented with affine transformations. The similarity metric used was the correlation as this metric is one of the most used metrics in intrapatient registration. The output for each method was the correlation value, the number of iterations and the processing time. With these results, we performed an exploratory data analysis (EDA),^(35)^ the results of which provided an overview of the data structure showing the amplitudes, asymmetries, location, possible outliers, etc. Then we produced a box‐plot graphic to observe the dispersion of correlation values (Fig. 4), noting the outliers (“*”). We also drew a bar‐line graphic with average processing time and number of iterations for each method (Fig. 5).

From Fig. 4, discarding the outlier points and comparing the median correlation values, we observe that the best correlation values are obtained with the TM, and the worst values with the SM.

In Fig. 5, we see that the average processing time of TDM and SM are much lower than the other two methods. The TM, due to the long processing time, is excluded from the selection of the best registration method. As the SM has the worst correlation values (see Fig. 4), it is also excluded from the selection of the best method.

The global results of the final correlation values, the average processing time and the number of iterations suggests that the TDM is a fast 3D registration method, even when compared with a method that uses an optimization technique (the pattern search technique).

### A.3 Normalized metrics in the presence of pulmonary tumor

To study the behavior of normalized similarity metrics when performing the registration of thoracic CT exams in the presence of lung tumors (some exams available to us did not have a tumor), we created an artificial spherical tumor with a diameter between 10 mm and 60 mm, and placed it inside the pulmonary region. Then, we applied random transformations and performed the registration of this exam with the original exam (without artificial tumor or random transformations). The random transformation (selected by the computer) was: translated by 1 or 2 voxels in each axis, rotated by −1° or −2°, scaled by a factor of 1.01 and sheared by a factor of −0.01. These procedures were applied to one exam from each patient, in a total of 20 patients; all metrics described in Section II.A.3 were computed. To reduce computational time, each exam was downsized from 512×512×n voxels to 128×128×n voxels.

**Figure 6:**
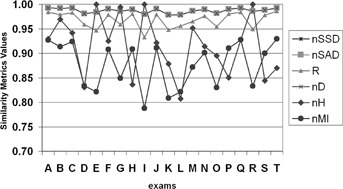
Values for all similarity metrics, for the registration of a CT exam with the same exam after inserting an artificial tumor with 60 mm diameter and applying random transformations.

For each metric, the starting value, the value after mass center alignment and the final value were computed, for a tumor of 60 mm of diameter, and also for a tumor of 30 mm of diameter (Fig. 6).

From Fig. 6, we see that the similarity metrics nSSD, nSAD and nD, have coincident values for all exams processed, suggesting that these metrics have equivalent behavior; this conclusion corresponds with the analysis of the curve shape and values of these metrics, represented in Fig. 2.

**Figure 7:**
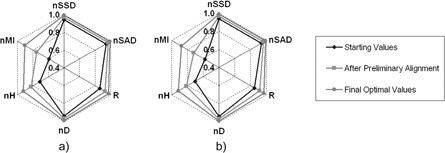
Average metric values for the registration of a CT exam with the same exam after inserting an artificial tumor with 30 mm (a) or 60 mm (b) of diameter and applying random transformations.

For a different analysis, we proceeded as previously described (creating an artificial spherical tumor, applying random transformations and performing the registration of this modified exam with original exam), computing for all metrics, the starting value (before applying any procedure), the value after preliminary alignment based on mass center of CT exam, and the final optimal value, for all 20 patients. Then, for each metric, we computed the average value for each group: starting values, the values after preliminary alignment, and the final optimal values. These computations were made with different tumor sizes. In Fig. 7, we include charts with results of registration with a tumor size of 30 mm and 60 mm.

We are searching for the normalized similarity metric with large dispersion values. In Fig. 7, we see that nMI is the metric with a large range of values. Even if we discard the interior polygonal line (that corresponds to the starting values) and compare only the range between values after preliminary alignment and the final optimal values, we observe that the nMI has the large interval with size of 0.156 (from 0.751 to 0.907 in the registration with a tumor of 30 mm diameter; from 0.720 to 0.876 in the registration with a tumor of 60 mm diameter). As all metrics are normalized, these results suggest that the nMI is the normalized similarity metric that is more sensitive to tumor changes.

### A.4 Normalized metrics in the registration of intrapatient CT exams

**Figure 8:**
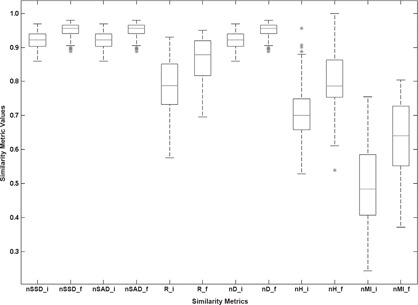
Box‐plot graphic for the comparison of six similarity metrics, before and after main registration procedure.

For the following analysis, we performed the registration of each exam with the remaining exams from the same patient, using the TDM, obtaining 300 combinations of intrapatient registration exams. The metric value after the preliminary alignment and the metric final optimal value were computed and a statistical analysis was made (Fig. 8).

In Fig. 8, a box‐plot graphic was drawn using 300 intrapatient registration combinations. In detail, we used all exams from Table 1 and performed the registration of each exam with all of the remaining exams from the same patient. For example, patient B (from Table 1) had three exams (B1, B2 and B3). We performed the registration of B1 with B2, B1 with B3 and B2 with B3. Patient K had 13 exams, which corresponds to (13!/[2!×(13−2)!])78 combinations for the intrapatient registration. Computing for all patients, we reached a total of 300 combinations for the intrapatient registration.

As some patients had a large number of CT exams (see patients K, L and M in Table 1), the registration of exams from these patients are predominant in the present statistical analysis. But, as the results extracted from this section will be compared with the previous section, we believe that final conclusions will not be strongly influenced by a very small number of patients, even when these patients had more than 10 CT exams.

For each registration, we computed the metric value after the preliminary alignment (represented in Fig. 8 with suffix “_i”, for initial) and the final optimal metric value (represented in Fig. 8 with suffix “_f”, for final). For comparison purposes, we discarded the outliers and compared the dispersion range of the second and third quartiles (the rectangular region for each metric), and also the four quartiles (the rectangular region plus the upper and lower branches).

In Fig. 8, we can observe that nSSD_f, nSAD_f and nD_f have the lower dispersion intervals (values from 0.888 to 0.979). When comparing only the second and third quartiles, we see that nMI_f has the large dispersion interval (values from 0.553 to 0.727). Comparing the four quartiles, we see that nMI_f has one of the largest dispersion intervals (values from 0.372 to 0.804), as well as nH_f (values from 0.611 to 0.999). However, the values of the second and third quartiles from nH_f (values from 0.753 to 0.862) are considerably smaller than nMI_f (values from 0.553 to 0.727). Comparing with the remaining similarity metrics in Fig. 8 and recalling the information extracted from Fig. 7, we conclude that nMI is the similarity metric that is more sensitive to the variation of tumor dimensions in the 300 intrapatient registrations performed from 20 patients.

In Table 2, we include the final optimal values of nMI, for some patients, after the registration of the last CT exam acquired with each remaining CT exams (for the same patient).

**Table 2:** nMI values from intra‐patient registration of last CT exam acquired with the remaining CT exams, for selected patients.

*Patients Versus Exams*	*T1*	*T2*	*T3*	*T4*	*T5*	*T6*	*T7*	*T8*
A	0.718	0.742	0.767	0.786
D	0.670	0.754	0.751	0.716	0.756
I	0.502	0.554	0.552	0.587	0.671
J	0.676	0.713	0.751	0.742
M	0.494	0.623	0.630	0.554	0.681	0.695	0.567	0.554

From Table 2, we observe that the nMI values for patient A are increasing with time, suggesting that tumor size is decreasing and the medical treatment applied to patient produces positive results. In the remaining patients, some have oscillating nMI values, suggesting that the medical treatment may not be reducing the tumors size or that patient is not receiving medical treatment. Other patients have results similar to the first patient of Table 2, corresponding to a reduction in their pulmonary tumors.

## IV. CONCLUSIONS

Present day computed tomography allows for volumetric data acquisition with very thin slices of the thoracic region, having a high resolution and contrast between lungs and nearby structures allowing for a detailed morphological analysis of pulmonary structures and tissues, with great importance to the diagnostic and follow‐up. An oncological patient may go through several tomographic acquisitions during a period of time resulting in several CT exams needing an appropriate registration before any follow‐up information can be extracted.

In this work, we analyze the behavior of several similarity metrics in the registration of intrapatient CT exams with pulmonary tumors. We propose an automatic 3D intrapatient registration method (TDM) and compare its performance with the traditional registration method ™ and also with optimized methods based on the pattern search optimization technique (PSM) and the simplex optimization technique (SM). The proposed method performs the segmentation of the lungs and builds a 3D image of the pulmonary region. Next, the center of mass is computed and the exams are coarsely aligned. Then, the 3D registration is performed using a downsized volume of the original CT exam. In spite of the fact that in some cases the PSM achieves very good results, our method is the best compromise between processing time and similarity metric values.

Six normalized similarity metrics are used in the intrapatient registration of pulmonary CT exams with lung tumors, and also in the intrapatient registration of the CT exams where artificial lung tumors were inserted. The results of these metrics are compared and allow us to conclude that the normalized mutual information is the best choice because it is more sensitive to the changes of tumor dimensions in CT exams.

The presented results with several cases of intrapatient intramodality registration show that the proposed registration method with the normalized mutual information metric is the best compromise between final metric values and processing time, which is needed for the quantitative tracking of lesions and the development of image fusion techniques that may effectively assist the follow‐up process of oncologic patients.
